# Hemocyte-mediated phagocytosis differs between honey bee (*Apis mellifera*) worker castes

**DOI:** 10.1371/journal.pone.0184108

**Published:** 2017-09-06

**Authors:** Eva Marit Hystad, Heli Salmela, Gro Vang Amdam, Daniel Münch

**Affiliations:** 1 Faculty of Chemistry, Biotechnology and Food Science, Norwegian University of Life Sciences, Aas, Norway; 2 Department of Biosciences, Centre of Excellence in Biological Interactions, University of Helsinki, Helsinki, Finland; 3 Faculty of Environmental Sciences and Natural Resource Management, Norwegian University of Life Sciences, Aas, Norway; 4 School of Life Sciences, Arizona State University, Tempe, Arizona, United States of America; Pusan National University, REPUBLIC OF KOREA

## Abstract

Honey bees as other insects rely on the innate immune system for protection against diseases. The innate immune system includes the circulating hemocytes (immune cells) that clear pathogens from hemolymph (blood) by phagocytosis, nodulation or encapsulation. Honey bee hemocyte numbers have been linked to hemolymph levels of vitellogenin. Vitellogenin is a multifunctional protein with immune-supportive functions identified in a range of species, including the honey bee. Hemocyte numbers can increase via mitosis, and this recruitment process can be important for immune system function and maintenance. Here, we tested if hemocyte mediated phagocytosis differs among the physiologically different honey bee worker castes (nurses, foragers and winter bees), and study possible interactions with vitellogenin and hemocyte recruitment. To this end, we adapted phagocytosis assays, which—together with confocal microscopy and flow cytometry—allow qualitative and quantitative assessment of hemocyte performance. We found that nurses are more efficient in phagocytic uptake than both foragers and winter bees. We detected vitellogenin within the hemocytes, and found that winter bees have the highest numbers of vitellogenin-positive hemocytes. Connections between phagocytosis, hemocyte-vitellogenin and mitosis were worker caste dependent. Our results demonstrate that the phagocytic performance of immune cells differs significantly between honey bee worker castes, and support increased immune competence in nurses as compared to forager bees. Our data, moreover, provides support for roles of vitellogenin in hemocyte activity.

## Introduction

The honey bee (*Apis mellifera*) is an important pollinator. The alarming decline of managed colonies has spurred much research into disease epidemics and mechanisms to counteract pathogens [1–3]. Highly social honey bees show a flexible division of labor among female workers that are all functionally sterile and assist the queen. After emergence, young workers typically start as nest bees that nurse the brood and other colony members, including the queen. After 2–3 weeks, workers can progress to outside tasks, and collect nectar, pollen and water (foragers) (see [4] and references therein). The physiological changes associated with worker caste progression from nurses to foragers include changes in their nutrient storage [5], hormonal regulation [6, 7], oxidative stress resilience [8], mortality and aging dynamics [5, 9–11] as well as in immune pathways [6, 9, 12]. Brood-less, over-wintering colonies are maintained by another worker caste—winter (*diutinus*) bees, which actively engage in thermoregulation and have the longest worker-lifespan (reviewed by [13]).

Bee colonies encounter a multitude of infectious pathogens, including bacteria (e.g. *Paenibacillus larvae* and *Melissococcus plutonius*), fungi (e.g. *Ascospaera apis*) and viruses (e.g. deformed wing virus) [1–3]. To fight pathogens, honey bees and other invertebrates depend on an innate immune system, and do not feature the acquired, antibody-mediated immune response of vertebrates. Several recent studies, however, show that immune priming occurs in insects (reviewed by [14]), including trans-generational immune priming ([15], reviewed by [16]). The innate immune system is comprised of cellular and non-cellular (humoral) response pathways. Briefly, the three lines of cellular immune responses are phagocytosis, encapsulation and nodulation [17, 18]. Relatively few studies in honey bees have assessed the cellular responses directly, e.g. by measuring hemocyte (blood cells) counts [9, 19], or by analyzing nodulation [20, 21] and encapsulation [22]. In contrast, research into honey bee immunity typically has focused on gene expression of antimicrobial peptides (humoral response) [23–25] and phenoloxidase activity, which is involved in both humoral and cellular responses [19, 26].

Phagocytosis is a rapid, cellular cleaning process of the hemolymph, where individual hemocytes engulf large particles, such as bacteria and dead cells [17, 27, 28]. Hemocyte-mediated phagocytosis has been confirmed in honey bees [29], but functional aspects have not been studied in detail, including how this process may vary between the worker castes.

Contrasting immune responses among worker castes revealed that forager bees have a higher number of pycnotic (suggestive of cell death) hemocytes compared to nurses, which coincides with lower vitellogenin (Vg) levels in foragers [9]. Vg is a protein involved in honey bee worker stress tolerance, behavior and immunity. Vg is considered as a donor of zinc that is necessary for immune system function [6]. Lack of zinc in honey bees induces pycnosis, which may explain the loss of functional hemocytes in foragers [6]. Vg has been found in many cell types and in different honeybee tissues. Vg often adheres to membrane structures [30], where it can protect cellular function via antioxidative [30, 31] and anti-inflammatory [30] actions. In several fish species, Vg also enhances the phagocytic activity of immune cells [32–34]. However, to our best knowledge any direct interaction between Vg and hemocytes has not been described in insects.

Circulating hemocytes in adult insects are formed in the hematopoietic glands at larval and pupal stages [35–37]. In adult insects, new hemocytes are typically recruited by mitosis [38, 39]. Similar to vitellogenin abundance, hemocyte counts in adult honey bees are effects of labor division and parasite infection, which supports an active regulation [6, 40, 41]. Yet, links between hemocyte recruitment and Vg are not yet identified in insects.

The aim of this study was, firstly, to establish an efficient approach for studying phagocytosis in honey bee workers. Secondly, we aimed at using such quantitative phagocytosis assay to explore phagocytic differences between three worker castes (nurses, foragers and winter bees), for which previous studies had already identified differences in immunocompetence [6, 9, 12, 41]. We use a bead-based and a dye-based assay measuring phagocytosis and incorporation. We test if co-inoculation with *Escherichia coli* can affect phagocytic rate in the worker bees, as it does in *Anopheles gambiae* and *Aedes aegypti* [39, 42, 43]. Furthermore, we test if Vg is directly associated with the hemocytes. Finally, we explore correlations between phagocytic activity or Vg levels, and the recruitment of new hemocytes by mitosis in the worker castes.

## Materials and methods

### Subjects

All tests were performed with honey bees (*Apis mellifera carnica* Pollmann) collected from apiaries of the Norwegian University of Life Sciences (Aas, Norway). To allow simultaneous testing of phagocytic rate and vitellogenin levels in summer and winter worker castes, we transferred winter bee colonies into a flight room (N_conolny_ = 2). This triggers the transition to summer behaviors, including nursing and foraging [10]. Indoor colonies experienced a day: night cycle set to 12h. Apart from artificial light sources, temperatures also changed at 12h intervals with 25°C and 17°C as maximum and minimum, respectively.

To establish tests that assess phagocytic activity, we exclusively used mature nurse bees with a chronological age between 10–20 days. In order to obtain nurse bees of known age, newly emerged bees (age = 1 day) were paint marked (Uni Posca, Tiverton, United Kingdom) on their thorax for later identification, and were returned to two replicate host hives. When they were between 10–20 days, mature nurses were identified based on their paint mark and by performing nursing activity, i.e. having their heads in cells of the brood comb. Nurse bees where collected from two replicate hives on two different days (N = 20 each sampling day).

To induce foraging activity, feeders with pollen and 30% sucrose were provided. At the time of collection, winter bees collected from outside locations (N_colony_ = 2) and flight room foragers (former winter bees) had a chronological age of at least 5 months. Foragers were identified by paint marks they had received when visiting feeders. A minimum foraging duration of 3 days was ensured, by re-collecting foragers at least 3 days after they had initially being marked in the flight room. Mature nurse bees in these tests were born in the flight room, and again had a chronological age of 10–20 days (see before for identification).

### Injection of phagocytic markers

For quantification of phagocytosis in honey bees we adapted two methods that have been established for other insects. One such approach includes the injection and hemocyte uptake of fluorescently labeled latex beads [27, 44–47]. Another is based on injecting the fluorescent dye CM-Dil, and has been successfully used in *Anopheles gambiae*, where the dye is incorporated in more than 70% of circulating phagocytic hemocytes [39, 43]. We prepared injection solutions with latex beads (0.5μm fluorescent carboxylate-modified polysterene latex beads, Sigma- Aldrich, Saint Louis, Missouri, USA) by dilution in Grace’s insect medium (1:5, Sigma- Aldrich, Saint Louis, Missouri, USA). The alternative CM-Dil solutions (Vybrant^®^ CM-Dil Cell-labeling solution, Molecular probes, Eugene, Oregon, USA) contained 0.75mM CM-Dil in Grace’s insect medium. According King and Hillyer 2012 [43] CM-Dil was prepared fresh and has been injected within 15 min after preparation.

After collection, mature nurse bees were chilled at 4°C and fixated with insect pins to a wax plate. Then, honeybees were injected with 1.5μL of either latex beads or CM-Dil solution. Following this, individuals injected with latex beads were kept in an incubator set at 32°C for 2h before hemolymph extraction. Incubation time was chosen based on published protocols and pilot test runs, to ensure that incubation periods are sufficient to induce robust phagocytosis. Bees injected with CM-Dil were also kept at 32°C. Yet, in line with available CM-Dil based protocols we have chosen a shorter incubation time of only 20min before hemolymph extraction [43].

After puncturing the abdomen between the fourth and fifth tergite with a sterile needle (31 G, Sigma Aldrich Saint Louis, Missouri, USA), approximately 3μL hemolymph per animal were extracted with 1μL Drummond micropipettes (Drummond Scientific Co/ Sigma- Aldrich, Schnelldorf, Germany). Collected hemolymph was suspended into 50μL collection buffer consisting of 70% Grace’s insect medium, 20% anticoagulant solution (98mM NaOH, 186mM NaCl, 1.7mM EDTA and 41mM citric acid, buffer pH 4.5) and 10% Fetal Bovine Serum [47].

### Imaging for qualitative assessment of phagocytosis

**Fig 1:**
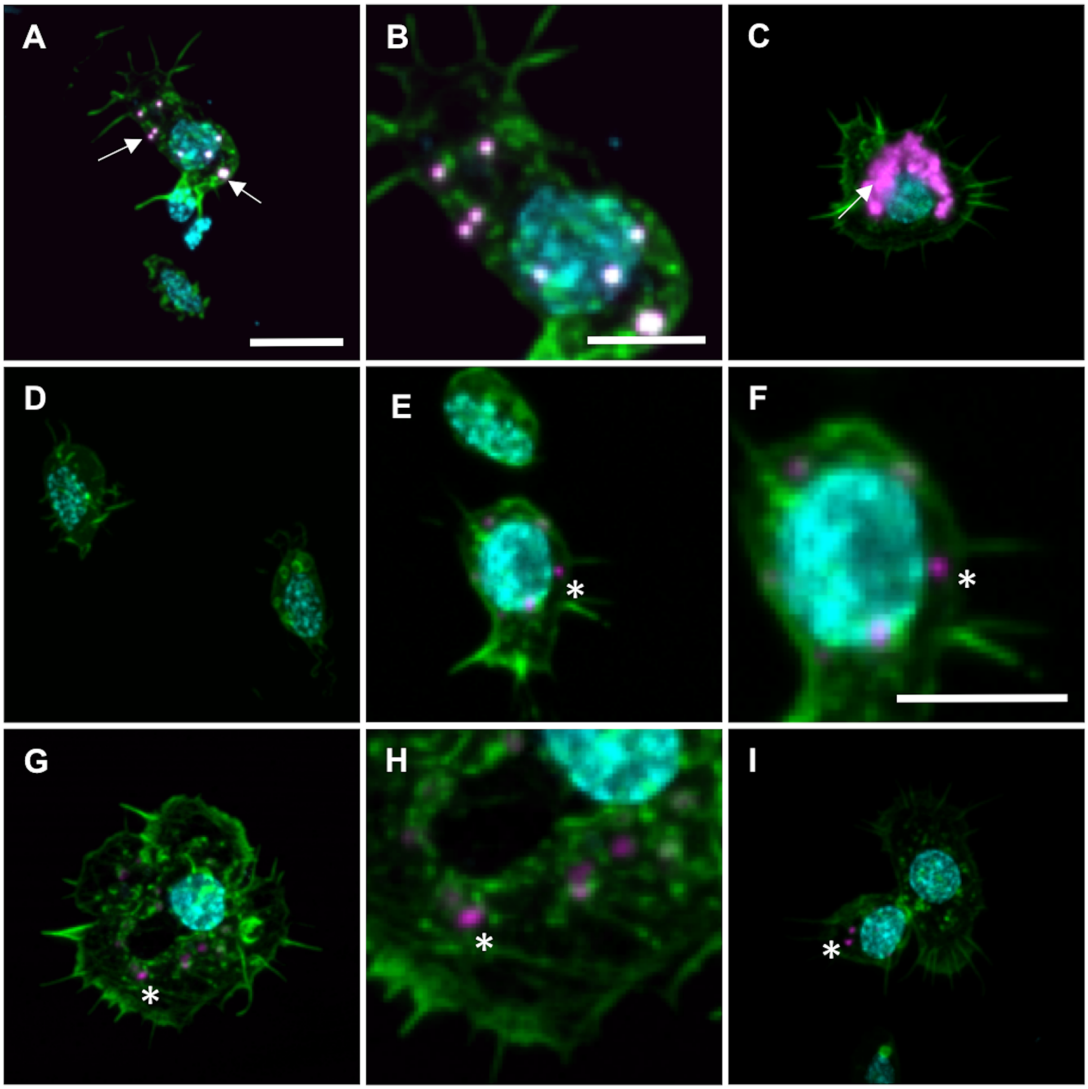
Circulating hemocytes from honey bee’s hemolymph take up the phagocytic markers; latex beads and CM-Dil. **A-C** Images showing hemocytes with latex beads (magenta, arrow) detected in the cytosol (labeled with phalloidin in green, nuclei labelled with DAPI in cyan). **B** is a zoomed image of the same hemocyte (plasmatocyte) detected in A. **C** Shows a hemocyte (granulocyte) that has internalized multiple latex beads. Such hemocytes were occasionally found. **D** An example of hemocytes without detectable phagocytosis of latex beads. **E-I** Hemocytes with detectable CM-Dil (arrow) in the cytosol (green, nuclei labeled with DAPI in cyan). Both F and H are zoomed images of E and G, respectively. **E** and **F** Shows a plasmatocyte. **G** Shows a granulocyte. **I** The upper cell to the right is an example of a hemocytes without detectable incorporation of CM-Dil, whereas the cell to the left show incorporation of CM-Dil (asterisks). Scale bar for A, C, D, E, G, I in A = 10μm, scale bar for B, F, H in B = 5μm, scale bar in F = 5μm.

High-resolution microscopic imaging was used to confirm phagocytosis and incorporation of latex beads and CM-Dil (compare Fig 1). For both treatments, the collected and re-suspended hemolymph was pipetted on poly-L-lysine coated slides. The slides were kept in an incubator at 32°C for 40min in order to let the hemocytes adhere to the slide. Subsequently, slides were fixed for 20min with 4% paraformaldehyde (PFA, Sigma-Aldrich, Saint Louis, Missouri, USA) dissolved in phosphate buffered saline (PBS, Sigma-Aldrich, Saint Louis, Missouri, USA). Cells were permeabilized for 5min in 0.2% Triton-X 100 in PBS and washed 3 times for 5min in PBS. Finally, samples were co-stained for 45min with the nuclear marker (4',6-diamidino-2-phenylindole, DAPI; 1:1,000 from 0.5mg/mL stock; Sigma-Aldrich, Saint Louis Missouri, USA), and a marker for the cellular matrix protein F-actin (Alexa Flour 488 Phalloidin; Molecular Probes, Eugene, Oregon, USA). After staining, slides were washed 3 times for 5 min before they were mounted in 30% glycerol/PBS, and were sealed with nail polish.

Confocal micrographs were acquired on a Leica TCS SP5 laser-scanning confocal microscope (Leica Microsystems, Wetzlar, Germany). High-resolution images were taken with a 40x immersion oil objective (numerical aperture 1.25). The simultaneous acquisition mode was used to minimize spatial shifts between color channels; the z-step size was kept at 0.5μm. Signal detection bandwidth differed slightly between latex beads (excitation = 561nm, detection = 580-630nm) or CM-Dil stain (excitation = 561nm, detection = 570-617nm). Settings for the remaining two channels were kept constant, independent of treatment, namely 405nm/430-480nm for excitation/detection of DAPI and 488nm/580-650nm for excitation/detection of Alexa Flour 488 Phalloidin. Image processing with the ImageJ software package [48] included maximum intensity projection of image stacks, and Gaussian filtering (kernel size = 1) to remove high spatial frequency noise.

Morphological information from imaging allowed us to classify hemocytes by using established classification systems. Based on detailed descriptions for several insects [18, 38, 49–52], we identified granulocytes, plasmatocytes and prohemocytes in our samples from honey bees.

### Activation of the immune system with *E*. *coli*

We tested if *E*. *coli* inoculation prior to marker injection can affect phagocytosis. To this end, we contrasted three treatment groups that all were injected with latex beads: one group with prior injection of 1μL of *E*.*coli*/Grace’s insect medium solution (strain ATTC 25404 K-12), a first control with prior injection of 1μL Grace’s insect medium only and a second control without prior injection. *E*.*coli* was grown overnight in Lysogeny broth (Sigma-Aldrich, Saint Louis, Missouri, USA) at 37°C on a shaker. Cell counts were diluted to approximately 800.000 per μL. Cells were centrifuged at 5200 rpm for 5min, washed twice and re-suspended to the same concentration in Grace’s insect medium. Prior to latex bead injection, honeybees were incubated for 2h. After latex bead injection, the honeybees were incubated for additional 2h as before, in total 4h.

### Imaging for quantifying phagocytosis

For quantitative analysis of microscopic images samples were handled essentially as before (previous section). Minor protocol differences include that for staining with DAPI only, no permeabilization with Triton-X was needed. A lower magnification objective (20x oil, numerical aperture 0.7) was used to acquire images with a larger overall dimension, and hence a greater number of hemocytes. To reduce technical bias in signal acquisition, laser power and sensor sensitivity settings were kept constant for all images. Five image replicates were taken for representing each individual; all cells in each image were counted, >50 cells were counted for each individual.

### Contrasting nurses, foragers and winter bees by high throughput quantification of phagocytosis

In order to measure phagocytosis with flow cytometry, individual foragers, nurse and winter bees were collected simultaneously and injected with latex beads (see before). After hemolymph extraction, the sample preparation was essentially the same, apart from the following modifications. Hemolymph was collected directly into tubes with 100μL collection buffer on ice. 100μL of 4% PFA and a 1:1000 dilution of DAPI in PBS was added to the hemolymph. Samples were analyzed using a MACSQuant^®^ analyzer, >3000 cells were counted for each individual (Miltenyi Biotec GmBH, Bergisch Gladbach, Germany). DAPI was detected using the V1 channel (excitation = 405nm, detection = 450nm), fluorescent latex beads were detected in the R1 channel (excitation = 635nm, detection = 655-730nm). Flow cytometry data were analyzed with the MASCQuantify^™^ software package (Miltenyi Biotec GmBH, Bergisch Gladbach, Germany). Negative controls for phagocytosis and hemocyte markers were included, by omitting latex bead and DAPI staining, respectively. Non-hemocyte particles were excluded by gating the cell population in forward scatter (FSC) vs side scatter (SSC). Gating adjustments to remove additional background noise, i.e. particles without DAPI or without latex beads, were made by using the negative control samples. The resulting data allowed to calculate the phagocytic rate by contrasting the counts for DAPI only objects (hemocytes), and objects that were both positive for DAPI and for the phagocytosis marker (hemocytes with latex beads).

Using flow cytometry, we could not distinguish the hemocyte types based on size and granularity dot plots, a typical approach used for blood testing. However, our results are in line with a previous study (de Graaf et al., 2002), that explains the failure to detect type dependent differences with the relatively small size of honey bee hemocytes.

### Imaging of Vg positive hemocytes

Hemolymph extraction and imaging was essentially done as described above in the section of qualitative assessment of phagocytosis. For Vg specific immunochemistry, (compare S1 Fig), slides with hemocytes were first blocked for 30min with 5% BSA/PBS, followed by overnight incubation with the rabbit-anti-Vg-antibody 1:500 (except for negative controls) at 4°C. Samples were again washed once in PBS for 5min and subsequently incubated with the secondary antibody (1:200 for 1h, Alexa-647 conjugated goat-anti-rabbit antibody in PBS-1% BSA). Finally, samples were co-stained for 45min with 1:1000 DAPI and Alexa Flour 488 Phalloidin. After staining, slides were washed 2 times for 5min before they were mounted and imaged as described before.

### Quantification of Vg positive hemocytes in nurses, foragers and winter bees

For quantification of Vg positive hemocytes, we contrasted nurses, foragers and winter bees. Hemolymph was collected directly into tubes with 100μL collection buffer on ice. 100μL of 4% PFA in PBS was added to the hemolymph and incubated at room temperature for 20min, followed by centrifugation at 2000g for 5min at room temperature. Cells were re-suspended in 0.2% Triton-X in PBS (5min) for permeabilization. This was followed by one washing with PBS, and blocking with PBS-5% bovine serum albumin (BSA). Samples were then incubated overnight at 4°C with a specific rabbit anti-Vg-antibody (1:500 in PBS-1% BSA, Salmela et al., 2015), again followed by 2 washes with PBS. Samples were then incubated for 20min with Alexa-647 secondary antibody. After three washes, samples were re-suspended in PBS together with a 1:1000 dilution of DAPI. Extensive washing of hemocytes typically results in a reduction of hemocyte numbers in the samples. Yet, at least 1000 events per each sample were recorded in the flow cytometric measurement. In the flow cytometer, DAPI was detected as before, Vg was detected in R2 (excitation = 635nm, detection = 750nm). Single color controls included a DAPI-only control, and a negative control for Vg, with only secondary antibody incubation, additionally, background noise was removed.

### Correlation analysis for phagocytic rate and Vg levels related to mitosis

Data on phagocytic rate and Vg levels were obtained from experiments described in the sections “Contrasting nurses, foragers and winter bees by high throughput quantification of phagocytosis” and “Quantification of Vg positive hemocytes in nurses, foragers and winter bees”, respectively. Mitosis levels were calculated based on the different DAPI-intensity distributions. Briefly, a more intense DAPI staining due to their greater amount of DNA and a second peak in the DAPI-signal histogram [53, 54], identifies mitotic cells.

### Statistical analyses

The T-test was used to test different phagocytic rate between CM-Dil and latex bead injections. Phagocytic rates in tests with *E*.*coli* pre-injection and two controls were contrasted using one-way ANOVA. Phagocytic rates and Vg levels in different worker types were analyzed by a one-way ANOVA, and post hoc Fisher Least Significant Difference (LSD) test. Possible links between phagocytosis and mitosis, as well as vitellogenin and mitosis were tested with Pearson’s correlation analysis. All analyses were performed with the Statistica 13.2 software package (Round Rock, TX, USA).

## Results

### Establishing phagocytic markers in honey bees

**Fig 2:**
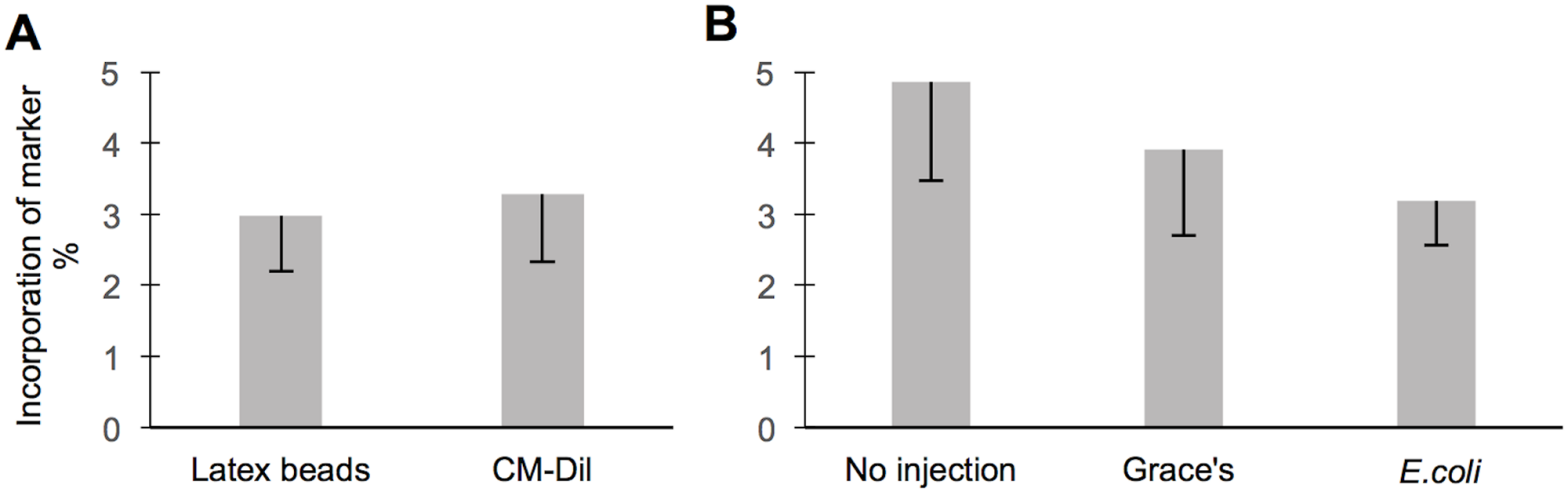
Measurements of phagocytic events do not differ between phagocytic markers and are not affected by a bacterial challenge. **A** Quantitative analyses of the latex bead and CM-Dil uptake with high-resolution microscopy yield similar results (T-test: *P*>0.05). The relative ratio of marker positive hemocytes in mature nurse bees is shown as mean ± SE. **B** Activation with *E*.*coli* or Grace’s insect media as control does not change the relative ratio of the phagocytic cells in mature nurse bees compared to the individuals with no activation, shown as mean ± SE.

To facilitate our studies, we established protocols for incorporation of two phagocytic markers in honey bees; fluorescent latex beads and CM-Dil stain. Microscopy confirmed that both markers could be incorporated by hemocytes (Fig 1). Using available morphological classification systems [18, 38, 49–52], we found that plasmatocytes and granulocytes were taking up both fluorescent markers (e.g., compare Fig 1A and 1C). Typically, the phagocytic cells took up only few latex beads (Fig 1A and 1B). Likewise, only a small fraction of the cells’ cytosol was found to be positive for the CM-Dil stain (Fig 1E–1I). In contrast, observations of the uptake of larger bead numbers by a single hemocyte was rare (Fig 1C). Similar for both labeling techniques, we found that about 3% of hemocytes were positive for the two markers (Fig 2A, T-test: N_latex beads_ = 11, N_CM-Dil_ = 15, F = 2.003, *P* = 0.27). This supports that latex beads and CM- Dil are both suitable as phagocytic markers. The data are presented in detail in S1 Table. Lastly, we tested if immune activation by *E*.*coli* 2h prior to latex bead injection affects the level of detectable phagocytosis. We found that phagocytosis levels were not different in individuals with prior *E*. *coli* injection and in the two controls, i.e. with prior Grace’s medium injection and no prior injection (Breakdown and one-way ANOVA: N_no injection_ = 9, N_grace’s medium_ = 12, N_*e*.*coli*_ = 12, F = 0.57, *P* = 0.57, Fig 2B). Hence, we concluded that relative phagocytosis levels in honeybees could be quantified using latex bead injection without a prior immune activation by *E*. *coli* inoculation. Data are presented in S2 Table.

### Comparing phagocytic activity in different worker castes

**Fig 3:**
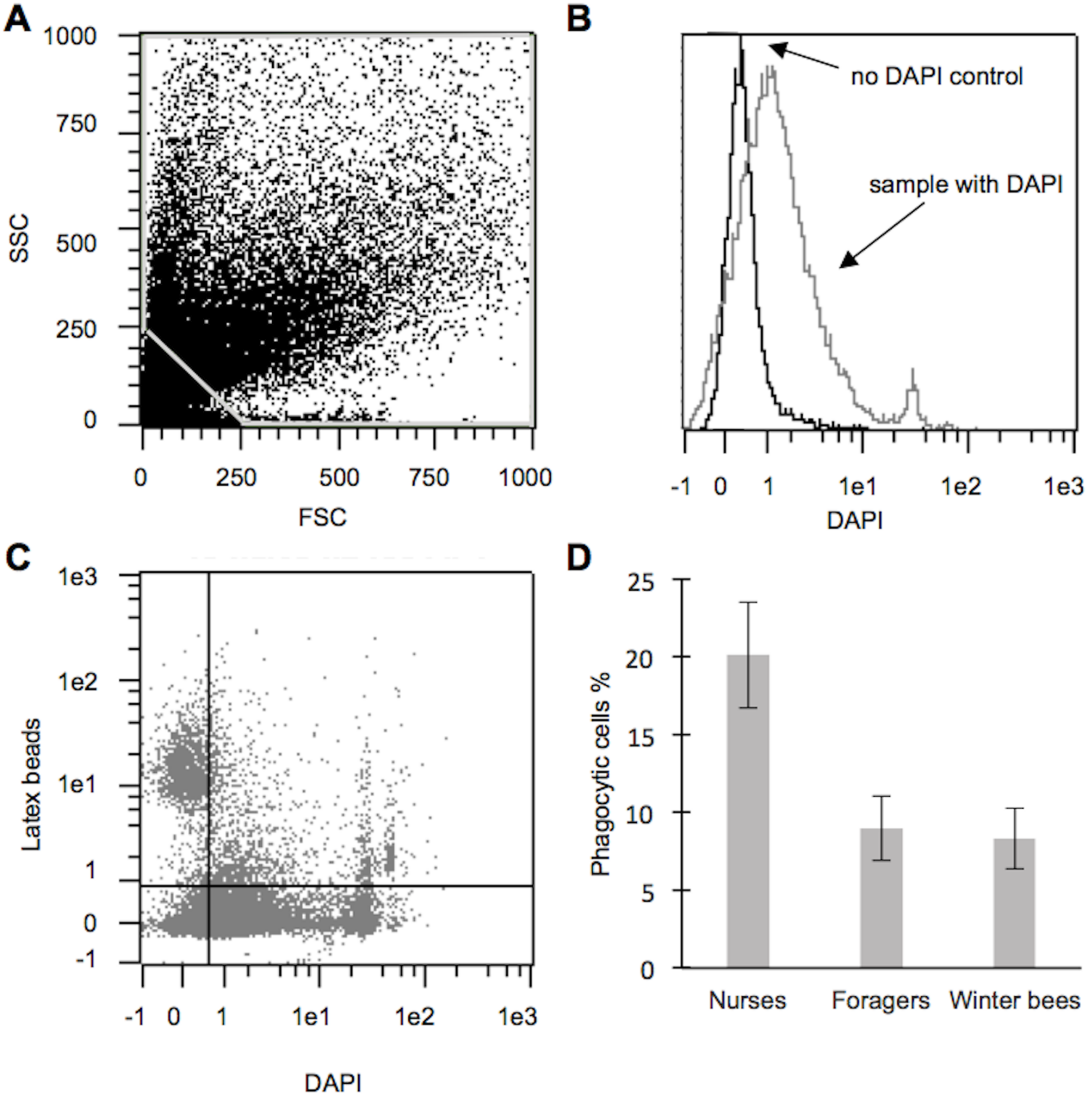
The phagocytic activity is higher in nurses than in foragers or winter bees. **A** Dot plot separated with FSC (size) and SSC (granularity) exemplifying the gating used to remove small particles and background noise. No clusters in hemocyte populations are detected. **B** A histogram of one hemocyte sample incubated with DAPI (grey), and a hemocyte control sample incubated without DAPI (black). The histogram illustrates how to exclude background and false positives from the DAPI-specific fluorescence. **C** Dot plot of latex beads *versus* DAPI. Each dot represents a single particle from gating in A. DAPI positive cells with nuclei are shown to the right in the plot. To the left DAPI negative particles are shown, which were excluded from further analysis (separated by the vertical line). Hemocytes in the upper right region are counted as phagocytic, and hemocytes in the lower right region as non-phagocytic (separated by the horizontal line). **D** Mature nurse bees have a statistically significantly higher percentage of phagocytic activity than foragers or winter bees (One-way ANOVA: *P*<0.01). Data are presented as means ± SEM.

We next established a flow cytometry assay for high-throughput analysis of phagocytosis in the honey bee. Honey bees were injected with latex beads—the phagocytic marker. The nuclear marker DAPI was used as a reference to count hemocyte numbers. No clear clustering of hemocyte population could be distinguished (Fig 3A for a representative example). The DAPI-labeled objects were further separated (gated) into phagocytic (co-labeled with DAPI and latex beads) or non-phagocytic (labeled only with DAPI) hemocytes (Fig 3C, compare Material and Methods section). Fig 3B shows the histogram for a DAPI labeled sample and a non-DAPI control.

We contrasted phagocytic activity in three different worker castes: mature nurse bees (n) and foragers (f) as well as winter bees (w). A flight-room was used to induce typical summer worker-type behavior, allowing the simultaneous collection of typical summer worker castes in the flight-room (nurses, foragers) and the winter worker caste at outside locations (winter bees). The average percentage of phagocytic hemocytes detected with flow cytometry was 20.1% in nurses, as compared to only 9.0% in foragers, and 8.3% in winter bees (see Discussion). We detected a significant difference in the phagocytic activity among worker castes (One-way ANOVA; N_n_ = 15, N_f_ = 13, N_w_ = 18, F = 6.8, *P*<0.01, Fig 3D). Post-hoc analyses revealed a significantly higher phagocytic activity in nurses as compared to foragers and winter bees (Fisher LSD test; *P*_(n vs f)_ < 0.01, *P*_(n vs w)_ < 0.01) but no significant difference between foragers and winter bees (*P*_(f vs w)_ = 0.85).

### Comparing levels of Vg in hemocytes in different worker castes

**Fig 4:**
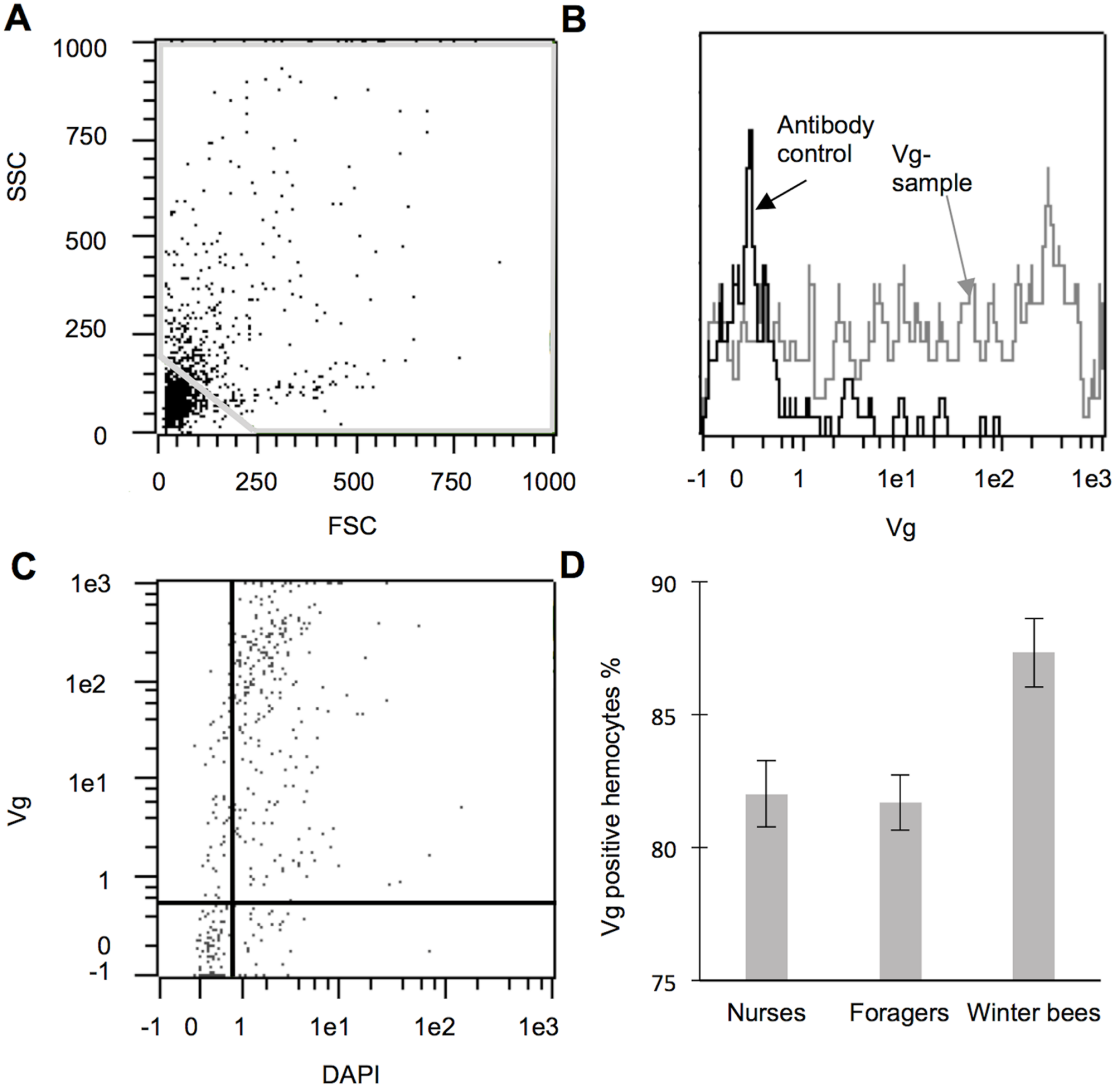
Quantification of Vg positive cells from hemolymph in nurses, foragers and winter bees. **A** A dot plot separated with FSC and SSC shows the gating for removing small particles and the background noise. **B** Immunostaining of hemocytes with and without the Vg primary antibody. The histogram shows hemocytes incubated with the secondary antibody only (control: black) and hemocytes incubated with both the Vg primary and secondary antibodies (grey). **C** Dot plot from a Vg positive sample, where the gating approach (the horizontal and vertical lines) are adapted from the controls of DAPI and Vg antibody. The two regions to the right are counted as hemocytes with the upper right region as Vg positive hemocytes. **D** The mean percentage of the Vg positive hemocytes is higher in winter bees compared to nurses and forager bees (One-way ANOVA: *P*<0.01). The data are presented as means ± SEM.

With high-resolution imaging, we established the presence of Vg within plasmatocytes and granulocytes (S1 Fig). We used flow cytometry to determine the number of Vg-positive cells in the hemolymph of nurse, forager and winter bees (Fig 4). The percentage of Vg- positive cells was calculated based on detected Vg and DAPI signals (compare previous section; Fig 4C). As illustrated in the histogram (Fig 4B), Vg-positive cells can be separated from Vg- negative objects by using a Vg-negative control as a reference. We found an overall effect of worker caste on the rate of Vg-positive hemocytes (One-way ANOVA; N_n=_ 14, N_f=_ 17, N_w=_ 14, F = 7.07, *P*<0.01). On average, winter bees had 87.3% Vg-positive hemocytes, while nurses and foragers had 82.0% and 81.7%, respectively (Fig 4D). Post hoc analyses revealed significantly higher levels of Vg positive hemocytes in winter bees compared to both nurse and forager bees (Fisher LSD test; *P*_(n vs f)_ = 0.84, *P*_(n vs w)_ <0.01, *P*_(f vs w)_ <0.001).

### Correlation analysis for phagocytic rate and Vg levels related to mitosis

**Fig 5:**
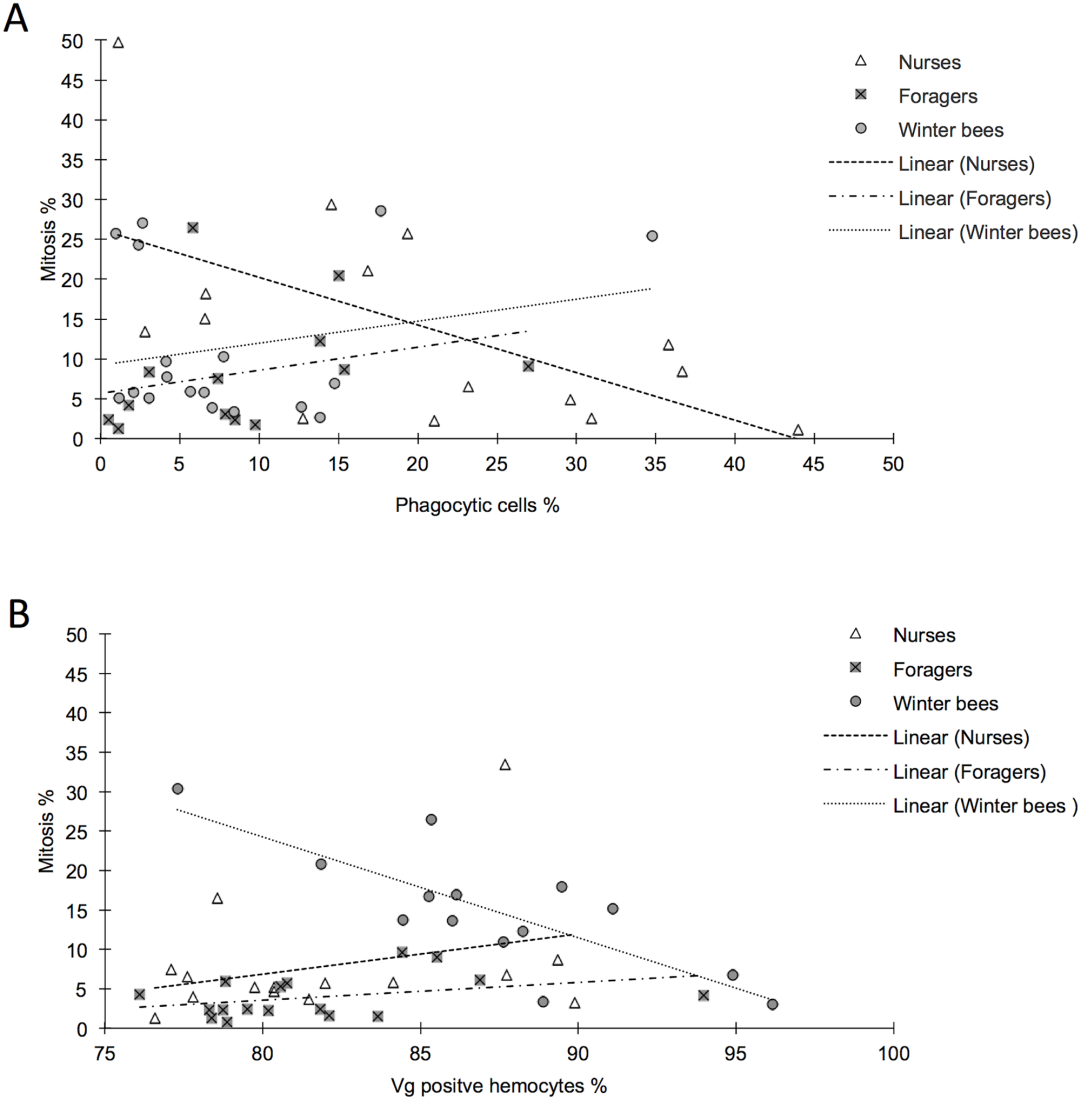
Correlation analysis, relation of mitosis and phagocytic hemocytes or vitellogenin positive hemocytes. **A** Phagocytic activity correlated to mitotic activity in nurses shows a significant negative correlation (r^2^ = 0.35, *P*<0.05). This indicates that an individual has either a high phagocytic activity or a high mitotic activity. In forager and winter bees, no correlation was detectable. **B** Winter bees show a negative correlation between Vg levels and mitotic activity (r^2^ = 0.63, *P*<0.001). This indicates that that the winter bees with a low number of vitellogenin positive hemocytes have more hemocytes that undergo mitosis. A significant correlation was not detected in nurse or forager bees.

We next studied possible correlations between phagocytosis and hemocyte recruitment through mitosis (Fig 5A; S3 Table), by using the same data set as for phagocytic rate assessments (compare Fig 3). For mature nurses, we found a significant negative correlation between phagocytic activity and mitotic activity (N = 15, r^2^ = 0.35, *P*<0.05). Foragers (N = 13, r^2^ = 0.08, *P* = 0.34) and winter bees (N = 18, r^2^ = 0.06, *P* = 0.34) showed no such correlation.

Lastly, we studied correlations between Vg levels and hemocyte mitosis (Fig 5B; S4 Table), using the same data set as for Vg in hemocytes assessments (compare Fig 4). For winter bees, we found a significant negative correlation between Vg levels and mitosis (N = 14, r^2^ = 0.63, *P*<0.001), while no relationships were detected in nurses (N = 15, r^2^ = 0.09, *P* = 0.28) and foragers (N = 17, r^2^ = 0.13, *P* = 0.15).

## Discussion

Here we confirm that honey bee worker castes differ in hemocyte function. Nurses show two-fold higher levels of phagocytic activity compared to long-lived winter bees and foragers. Moreover, our data support tradeoffs between phagocytic activity and hemocyte recruitment through mitosis. Lastly, we demonstrate that the majority of hemocytes are immunopositive for Vg, and that Vg levels are negatively correlated with mitotic activity. However, links between phagocytic activity, hemocyte recruitment and Vg levels were only detected for specific worker castes.

To assess phagocytic activity, we adapted protocols for different phagocytosis markers, which were established in other insect species, e.g., *D*. *melanogaster* and *A*. *gambiae* [43–47]. Our simultaneous use of the respective markers allowed us compare their performance with quantitative assays. Our results show that both CM-DiI and latex beads are taken up at similar rates independent of protocol differences, including greatly different incubation times (latex beads = 2h, CM-Dil = 20min). In addition, we tested if prior bacterial infection can increase phagocytic immune response [55] as shown for *A*. *Aegypti and A*. *gambiae* [39, 42, 43]. However, our data do not support a priming-effect for honey bee nurses at 4h after injection.

Comparing phagocytosis levels among different study systems or species is complicated by the use of different quantification methods. Similarly, with microscopy, we found incorporation of the markers to be approximately 3%, for both CM-Dil and latex beads, while flow cytometry yielded a considerably higher phagocytic rate of 20% (in nurses). This could indicate that phagocytic levels might have been underestimated using microscopy due to different sample preparation approaches [50]. For microscopic sample preparations, a critical step is adherence of hemocytes to the poly-L-lysine coated glass slides, hence that they are not washed away during staining procedures. Nevertheless, based on our flow-cytometry data for phagocytosis we found no support for the notion that honey bees possess a generally less competent immune-defense than other insects, although it is hard to compare values between studies. Previous studies in other species report both higher [43, 46], similar [45] and lower phagocytic rates [46] than we established with flow cytometry (8–20%) for honey bees. Perhaps different phagocytic rates are linked to specie-specific body size, with the smallest insects having the highest phagocytic activity [46].

An interaction between Vg and hemocytes was proposed a decade ago [6]. We detected Vg in the hemocytes of all worker castes tested, in both the plasmatocytes and granulocytes. The role of Vg in hemocytes might be related to its function as a zinc donor [6]. The zinc ion has important regulatory and catalytic roles in the immune system (reviewed by [56]). Another potential role for hemocyte-Vg is suggested by the finding that honey bee Vg acts as an opsonin that in the case of fish-Vg can make pathogens more susceptible to phagocytosis [32–34]. Specifically, honey bee Vg binds to surface molecules of pathogens, such as lipopolysaccharides [15], and this might activate hemocytes. Concerning worker-caste differences, we found highest levels of hemocyte-Vg for winter bees, reflecting the extreme Vg levels shown previously for total hemolymph fractions [57] reviewed by [13]. Despite the fact that foragers usually have lower Vg hemolymph levels than nurses [9], we found no difference in the number of Vg positive cells between these summer worker castes. A possible explanation for this could be that Vg, once internalized, does not degrade in persisting hemocytes.

The negative correlation between phagocytosis and mitosis we found for nurse bees, suggests a tradeoff, in which individuals with an already high phagocytic activity recruit less hemocytes. In turn, individuals with less phagocytic active hemocytes might need to recruit more hemocytes, and therefore increase mitotic activity. Yet, we could not find support for the latter, as reduced phagocytic activity in foragers did not correlate with mitotic activity. Likewise, winter bees showed no correlation between phagocytosis and mitosis, but a negative correlation between Vg levels and mitosis. This again may support that if protective mechanisms are already in place, like the abundant availability of Vg (compare [31]), there is less need to further increase immunocompetence, for example by mitotic hemocyte recruitment.

Based on available research data as well as on evolutionary theories on aging and cast-specific survival capacity (e.g., disposable soma theory), it has often been suggested that somatic maintenance is reduced in foragers. This view is supported by observations of reduced stress-resilience and faster aging in foragers [5, 10, 31, 58]. Along the same lines, we show that phagocytic levels are reduced in foragers as compared to nurse bees. This is in line with previous reports that found increased levels of pycnotic hemocytes in foragers [9], and that the nodulation response is abolished in forager bees [20]. Together, our data and the previous findings indicate a gradual loss of immune function integrity in foraging honey bees. Contrasting this conclusion are findings that show an increased phenoloxidase activity in older forager bees as compared to newly emerged bees [12, 41], or similar encapsulation responses in foragers and newly emerged bees [41]. A possible explanation for such contrasting findings is that newly emerged honey bees are immature. They perform poorly in many assays, including tests of sensory performance [59], learning and memory (discussed by [59]), flight ([60], for a general review on honey bee development see [61] and discussed by [62]), and worker physiology takes days to mature [61]. From this perspective, it may not be surprising that some aspects of honey bee immune function perform less well at the beginning of adult life.

## Supporting information

S1 Fig Vitellogenin is detectable within hemocytes. Both granulocytes and plasmatocytes were Vg positive. A-C Granulocyte morphology was identified by combined nuclear (DAPI, cyan) and F-actin (Alexa Flour 488 Phalloidin, green) staining. Superposition of the cell staining and the Vg (Alexa-647 conjugated goat-anti-rabbit antibody, red) signal suggests Vg to be localized within granulocytes (C). Scale bar for A-C = 10μm D-F Plasmatocyte morphology was labeled as in A-C and show co-localization with the Vg signal. Scale bar for D-F = 10μm.
(PDF)

S1 Table Data for comparison of beads and CM-Dil injection, Fig 2A. Percentage of incorporation of markers.
(PDF)

S2 Table Data for *E*.*coli* injection, Fig 2B. Percentage phagocytosis for the different individuals in the sample set.
(PDF)

S3 Table Data for phagocytosis and mitosis, Figs 3 and 5. Percentage phagocytosis and mitosis for the different individuals in the sample set.
(PDF)

S4 Table Data for vitellogenin levels and mitosis, Figs 4 and 5. Percentage of vitellogenin levels and mitosis for the different individuals in the sample set.
(PDF)
