# Supplementary figures and images for: Hemocyte-mediated phagocytosis differs between honey bee (Apis mellifera) worker castes
Source: PLoS One. 2017 Sep 6;12(9):e0184108. doi: 10.1371/journal.pone.0184108 (PMC5587260; doi:10.1371/journal.pone.0184108)

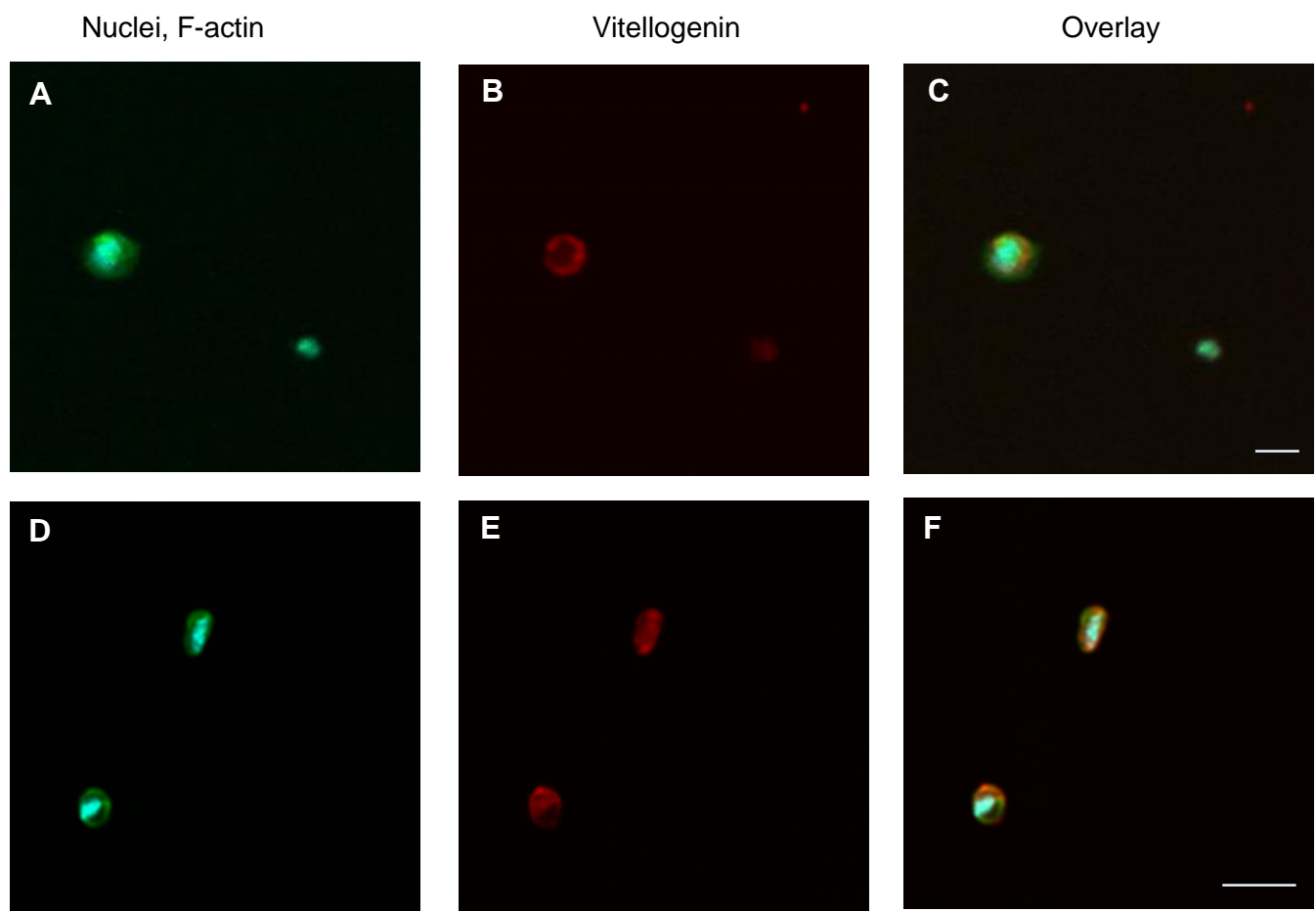

**S1 Fig**

Supplement: S1 Fig — A-C Granulocyte morphology was identified by combined nuclear (DAPI, cyan) and F-actin (Alexa Flour 488 Phalloidin, green) staining. Superposition of the cell staining and the Vg (Alexa-647 conjugated goat-anti-rabbit antibody, red) signal suggests Vg to be localized within granulocytes (C). Scale bar for A-C = 10μm D-F Plasmatocyte morphology was labeled as in A-C and show co-localization with the Vg signal. Scale bar for D-F = 10μm. (PDF) [file pone.0184108.s001.pdf]
