# Supplementary material for: Hemocyte-mediated phagocytosis differs between honey bee (Apis mellifera) worker castes
Source: PLoS One. 2017 Sep 6;12(9):e0184108. doi: 10.1371/journal.pone.0184108 (PMC5587260; doi:10.1371/journal.pone.0184108)
Supplement: S1 Table — Percentage of incorporation of markers. (PDF) [file pone.0184108.s002.pdf]

| ID | Percentage |
|---|---|
| Bead 1 | 1,851851852 |
| Bead 2 | 4,838709677 |
| Bead 3 | 1,923076923 |
| Bead 4 | 1,176470588 |
| Bead 5 | 5,970149254 |
| Bead 6 | 8,474576271 |
| Bead 7 | 0 |
| Bead 8 | 0 |
| Bead 9 | 3,125 |
| Bead 10 | 3,846153846 |
| Bead 11 | 1,650165017 |
| CM-Dil 1 | 4,87804878 |
| CM-Dil 2 | 8,421052632 |
| CM-Dil 3 | 4,47761194 |
| CM-Dil 4 | 1,408450704 |
| CM-Dil 5 | 13,63636364 |
| CM-Dil 6 | 4 |
| CM-Dil 7 | 1,612903226 |
| CM-Dil 8 | 3,496503497 |
| CM-Dil 9 | 0 |
| CM-Dil 10 | 0 |
| CM-Dil 11 | 0 |
| CM-Dil 12 | 0 |
| CM-Dil 13 | 1,515151515 |
| CM-Dil 14 | 3,719008264 |
| CM-Dil 15 | 2,127659574 |
