# Supplementary material for: Hemocyte-mediated phagocytosis differs between honey bee (Apis mellifera) worker castes
Source: PLoS One. 2017 Sep 6;12(9):e0184108. doi: 10.1371/journal.pone.0184108 (PMC5587260; doi:10.1371/journal.pone.0184108)
Supplement: S2 Table — Percentage phagocytosis for the different individuals in the sample set. (PDF) [file pone.0184108.s003.pdf]

Sample	% Phagocytic cells
Ec1	0
Ec3	4,109589041
Ec4	3,80952381
Ec5	1,639344262
Ec6	4,166666667
Ec7	0
Ec8	4,615384615
Ec9	3,968253968
Ec10	6,52173913
Ec12	0,760456274
Ec13	5,714285714
Ec14	2,941176471
Grace1	4,081632653
Grace2	0
Grace3	3,225806452
Grace4	0,492610837
Grace5	2,597402597
Grace6	14,70588235
Grace7	1,351351351
Grace8	4,761904762
Grace9	1,176470588
Grace10	8,379888268
Grace11	5,194805195
Grace12	0,892857143
Control 1	6,790123457
Control 2	13,63636364
Control 4	1,694915254
Control 5	0
Control 6	5,309734513
Control 7	1,986754967
Control 8	2,941176471
Control 9	3,448275862
Control 10	7,894736842
