# Supplementary material for: Hemocyte-mediated phagocytosis differs between honey bee (Apis mellifera) worker castes
Source: PLoS One. 2017 Sep 6;12(9):e0184108. doi: 10.1371/journal.pone.0184108 (PMC5587260; doi:10.1371/journal.pone.0184108)
Supplement: S3 Table — Percentage phagocytosis and mitosis for the different individuals in the sample set. (PDF) [file pone.0184108.s004.pdf]

ID	% phagocytic cells	% mitotic cells	Group
1 nurse h1.012	44,00452489	1,131221719	N
2 nurse h1 .014	12,77161863	2,527716186	N
3 nurse h1 .015	23,17229585	6,512754738	N
4 nurse h1 .016	36,69631512	8,411689962	N
7 nurse h2 .017	21,04956268	2,274052478	N
8 nurse h2 .019	35,78750293	11,84179733	N
10 nurse h2 .020	29,63385541	4,849082045	N
13 forager h1.022	0,49045367	2,355929235	F
14 forager h1.023	7,364173512	7,493875198	F
15 forager h1.024	1,076320939	1,204275177	F
19 forager h2 .028	3,031578947	8,392982456	F
21 forager h2 .029	15,02093643	20,41872859	F
22 forager h2 .030	13,84998223	12,22182723	F
25 winter h3.033	34,79566523	25,42107325	W
26 winter h3.034	2,659848947	27,03945208	W
27 winter h3.035	0,966414126	25,70542266	W
28 winter h3.036	17,63929768	28,53445651	W
31 winter h4 .039	6,519224541	5,792809883	W
32 winter h4 .040	7,006058833	3,875458679	W
33 winter h4 .041	8,416626192	3,387100897	W

34 winter h4 .042	13,82427223	2,635251894	W
37 nurse h2.045	19,3269668	25,69349704	N
39 nurse h1 .047	30,93795888	2,560572687	N
40 nurse h1 .048	1,07809131	49,74219556	N
42 nurse h1 .050	14,56568676	29,38050158	N
43 nurse h1 .051	2,797001153	13,45155709	N
45 nurse h2 .053	6,558122612	15,03547389	N
46 nurse h2 .054	16,83753258	21,04257168	N
47 nurse h2 .055	6,630921678	18,16207411	N
49 forager h1 .057	9,726463104	1,692111959	F
51 forager h1.059	5,791106515	26,4506492	F
52 forager h1.060	15,38082933	8,687092408	F
53 forager h1.061	7,836485455	3,048935629	F
54 forager h1.062	1,741877642	4,216653147	F
55 forager h1.063	8,478292647	2,368074843	F
56 forager h1.064	26,96502058	9,063786008	F
58 winter h3.066	12,67605634	3,979031137	W
59 winter h3.067	3,049488235	5,070169885	W
60 winter h3.068	2,380170716	24,35160867	W
61 winter h3.069	7,741303283	10,32990364	W
62 winter h3.070	5,669761273	5,868700265	W

65 winter			
h4 .073	4,183311977	7,786236527	W
66 winter			
h4 .074	14,7611486	6,894270213	W
67 winter			
h4 .075	2,06779998	5,835570136	W
68 winter			
h4 .076	1,154912227	5,043116723	W
69 winter			
h4 .077	4,107142857	9,62406015	W
