# Supplementary material for: Hemocyte-mediated phagocytosis differs between honey bee (Apis mellifera) worker castes
Source: PLoS One. 2017 Sep 6;12(9):e0184108. doi: 10.1371/journal.pone.0184108 (PMC5587260; doi:10.1371/journal.pone.0184108)
Supplement: S4 Table — Percentage of vitellogenin levels and mitosis for the different individuals in the sample set. (PDF) [file pone.0184108.s005.pdf]

Name	% Vg positive cells	% Mitotic cells	Group
_1 N.014	89,87730061	3,269754768	N
_2 N.015	81,4516129	3,645833333	N
_3 N.016	87,71929825	6,707317073	N
_4 N.017	81,97278912	5,685618729	N
_7 N.018	89,33717579	8,707865169	N
_8 N.019	76,57657658	1,307189542	N
_9 N.020	77,77777778	3,95480226	N
_10 N.021	79,75206612	5,185185185	N
_13 F.022	84,40860215	9,578544061	F
_14 F.023	79,50819672	2,43902439	F
_15 F.024	80,56338028	5,292479109	F
_16 F.025	93,97260274	4,146341463	F
_19 F.026	80,76923077	5,703422053	F
_20 F.027	78,31325301	2,325581395	F
_21 F.028	86,8852459	6,111111111	F
_22 F.029	82,11382114	1,621621622	F
_25 W.030	86,14457831	16,91729323	W
_26 W.031	91,08910891	15,15151515	W
_27 W.032	94,90333919	6,692913386	W
_28 W.033	86,02150538	13,63636364	W
_31 W.034	88,23529412	12,32876712	W
_32 W.035	89,47368421	17,92828685	W
_33 W.036	96,16724739	3,006329114	W
_34 W.037	88,88888889	3,344481605	W
_37 N.054	87,67908309	33,43108504	N
_38 N.055	77,09251101	7,5	N
_39 N.056	84,14096916	5,853658537	N
_43 N.057	78,57142857	16,52542373	N
_44 N.058	80,3652968	5,240174672	N
_45 N.059	80,35320088	4,705882353	N
_50 F.061	78,86178862	0,787401575	F
_51 F.062	78,37837838	1,276595745	F
_55 F.063	85,5227882	9,034267913	F
_56 F.064	78,82736156	5,94795539	F
_57 F.065	78,74015748	2,272727273	F
_61 W.066	77,28842832	30,42328042	W
_62 W.067	85,2739726	16,72473868	W
_63 W.068	85,34883721	26,50918635	W
_67 W.069	87,6146789	10,93023256	W
_68 W.070	81,84931507	20,81784387	W
_69 W.071	84,43579767	13,75	W
_FA H1.038	80,17241379	2,162162162	F
_FB H1.039	83,65384615	1,449275362	F
_FC H1.041	76,10619469	4,301075269	F
_FD H1.042	81,81818182	2,4	F
_NA H1.072	77,6119403	6,52173913	N
